# Physical Exercise Is Confirmed to Reduce Low Back Pain Symptoms in Office Workers: A Systematic Review of the Evidence to Improve Best Practices in the Workplace

**DOI:** 10.3390/jfmk4030043

**Published:** 2019-07-05

**Authors:** Stefano Gobbo, Valentina Bullo, Manuele Bergamo, Federica Duregon, Barbara Vendramin, Francesca Battista, Enrico Roma, Danilo Sales Bocalini, Roberta Luksevicius Rica, Cristine Lima Alberton, David Cruz-Diaz, Giampaolo Priolo, Vittorio Pancheri, Stefano Maso, Daniel Neunhaeuserer, Andrea Ermolao, Marco Bergamin

**Affiliations:** 1 Sport and Exercise Medicine Division, Department of Medicine, University of Padova, 35128 Padova, Italy; 2 Laboratorio de Fisiologia e Bioquimica Experimental, Centro de Educacao Fisica e Deportos, Universidade Federal do Espirito Santo (UFES), SP 01504-00 Vitoria, ES, Brazil; 3 Departamento de Educacao Fisica e Ciencias do Envelhecimento, Laboratorio de Percepcao Corporal e Movimento, Universidade Sao Judas Tadeu, 03166-000 Sao Paulo, SP, Brazil; 4 Department of Sports, Physical Education School, Federal University of Pelotas, 96055630 Pelota, RS, Brazil; 5 Department of Health Sciences, Faculty of Health Sciences, University of Jaén, E-23071 Jaén, Spain; 6 Freelance Professional and Occupational Physician, Lungargine Panvinio 31, 37121 Verona, Italy; 7 Freelance Professional and Occupational Physician, Viale Venezia 87/A, 35015 Conegliano, Italy; 8 Department of Cardiac, Thoracic and Vascular Sciences, University of Padova, 35128 Padova, Italy

**Keywords:** workplace, exercise, low back pain, review, office worker

## Abstract

This systematic review aimed to analyze the effects of a physical exercise (PE) program on low back pain (LBP) symptoms of office workers and the modification of flexibility and range of motion (ROM), muscular strength, and quality of life (QoL). A literature research was performed on PubMed, Scopus, MEDLINE, and SPORTDiscus from April to May 2018. The keyword “low back pain” was associated with “office worker” OR “VDT operators” OR “office employees” OR “workplace” AND “exercise”, OR “exercise therapy” OR “physical activity”. Inclusion criteria were a home- or work-based exercise protocol for office workers with LBP symptoms and pre- to post-intervention evaluation of LBP symptoms. Three researchers independently examined all abstracts. The modified Cochrane methodological quality criteria were used for quality assessment and 11 articles were included. Exercise protocols were performed from 6 weeks to 12 months, 1–5 day per week, lasting 10–60 min for each session. Physical Exercise in the workplace improved all the considered outcomes. The best improvement was recorded in supervised protocols and in video-supported protocols performed in the workplace. The effect may be generated with small duration sessions during the working day, with only 10–15 min of adapted exercise to be performed 3–5 days per week.

## 1. Introduction

Low back pain (LBP) is a common worldwide disorder defined as a muscular tension, stiffness or pain localized below the rib margin and above the inferior gluteal folds, involving or not the leg [1]. Non-specific LBP is defined as pain without a known cause, representing 90–95% of cases, and the prevalence rate is approximately 18% [2]. Individuals with LBP display fear, anxiety, and disinformation about LBP. To avoid disability, individuals should continue ordinary activities depending on their pain management, returning to work as soon possible and avoiding rest positions [3]. The onset of LBP in the workplace can be caused by different occupational hazards such as repetitive bending and lifting (e.g., nurses, construction workers), vibrations (e.g., drivers), and long durations in standing or sitting positions (e.g., salespersons, office workers) [4]. In office workers, the prevalence of LBP is approximately 34% [5]. Risk factors are associated with prolonged static positions and psychosocial problems that increase the risk of developing chronic LBP and disability. Generally, individuals with LBP have negative attitudes toward pain, and tend to reduce their daily activity due to the wrong conviction that passive treatment will be beneficial [6]. Instead, physical exercise (PE) is strongly recommended for the management of LBP [3] as a prevention treatment [7]. Indeed, idiopathic LBP is usually associated with low levels of physical activity independent of pain-related disability. Physical exercise in the workplace is becoming a point of interest for companies and corporations in promoting a healthy lifestyle and improving the quality of life for their workers. In fact, PE should be increased for the prevention of LBP, including as treatment for several types of work-related LBP [8]. Moreover, it seemed that more active employees are more productive, requiring less sick leave, and having overall lower healthcare costs [9]. However, to the best of our knowledge, specific physical exercise recommendations for office workers are not commonly practiced, and occupational doctors do not commonly deliver specific exercise prescriptions for LBP. Therefore, the aim of this systematic review was to evaluate the current literature regarding exercise protocols developed for the work environment for the management of LBP symptoms in office workers.

## 2. Materials and Methods 

### 2.1. Study Design

The aim of this systematic review was to analyze the effects of PE on LBP symptoms, the modification of flexibility and range of motion (ROM), muscular strength, and quality of life of office workers. The Preferred Reporting Items for Systematic Reviews and Meta-Analyses (PRISMA) guidelines and flow chart diagram were used as a reporting structure [10].

### 2.2. Literature Research

The literature search and article revision were performed from April to May 2018. The keyword “low back pain” was associated with “office worker” OR “VDT operators” OR “office employees” OR “workplace” AND “exercise”, OR “exercise therapy” OR “physical activity”. The research was carried out on the online database PubMed, Scopus, MEDLINE and SPORTDiscus; no time restrictions regarding publication year were applied. In addition, reference lists for the included studies were screened.

### 2.3. Inclusion and Exclusion Criteria

Only studies published in English in peer-reviewed journals were considered eligible. To be included, articles had to meet the criteria in the Participants, Interventions, Comparators, Outcomes, Study (PICOS) model, as shown in Table 1 [10].

The inclusion criteria were a) male and female office workers with LBP symptoms; b) exercise protocol for LBP management; and c) evaluation of LBP symptoms. All studies not evaluating outcomes through pre- and post-intervention comparisons, as well as cross-sectional studies, reviews, commentaries, perspective studies, editorials, and case reports were then excluded. Published abstracts, dissertation materials, or conference presentations were not considered eligible documents. 

### 2.4. Study Quality Assessment

The quality of the studies was assessed by applying an adapted nine-criteria checklist provided by the Cochrane Collaboration Back Review Group [11]. As in previous systematic reviews, the checklist had to be marginally adapted to rate the strength of the evidence [12,13,14]. Each study in the review was scored on the basis of the following nine criteria: (1) “Was the method of randomization adequate?”; (2) “Were the groups similar at baseline regarding the outcome measures?”; (3) “Were inclusion and exclusion criteria adequately specified?”; (4) “Was the drop-out ratio described adequately?”; (5) “Were all randomized participants analyzed in the group to which they were allocated?”; (6) “Was compliance reported for all groups?”; (7) “Was intention-to-treat analysis performed?”; (8) “Was the timing of outcomes assessment similar in all groups?”; (9) “Was a followed-up performed?” 

If the paper provided a satisfactory description, a positive value was assigned (+). If the criterion description was considered absent, unclear, or lacked the specified content, a negative value was assigned (−). A study was qualitatively assessed as high quality if it showed a positive score in 5 out of 9 of the criteria, otherwise, it was considered a low-quality study.

### 2.5. Data Extraction and Synthesis

To perform an initial selection, three researchers independently examined all abstracts resulting from the literature search. Then, the full texts were read to include them in the revision process. Finally, the reference sections of the included items were screened to identify additional articles. Independent searches were then combined, compared, and reviewed to identify the included studies. In the case of discrepancies, a fourth researcher was consulted. A K-Cohen’s coefficient of 0.74 indicated a good agreement between researchers. Quality assessment using the modified Cochrane methodological quality criteria was then independently applied by the three researchers and discussed before the final quality scores assignation (Table 2). The same researchers who screened the titles, abstracts, full texts, and references performed quality assessments. Several domains were identified for categorization of the study results. In particular, studies were analyzed in regard to “pain and disability”, “flexibility, range of motion (ROM) and muscular strength”, and “quality of life”.

## 3. Results

**Figure 1:**
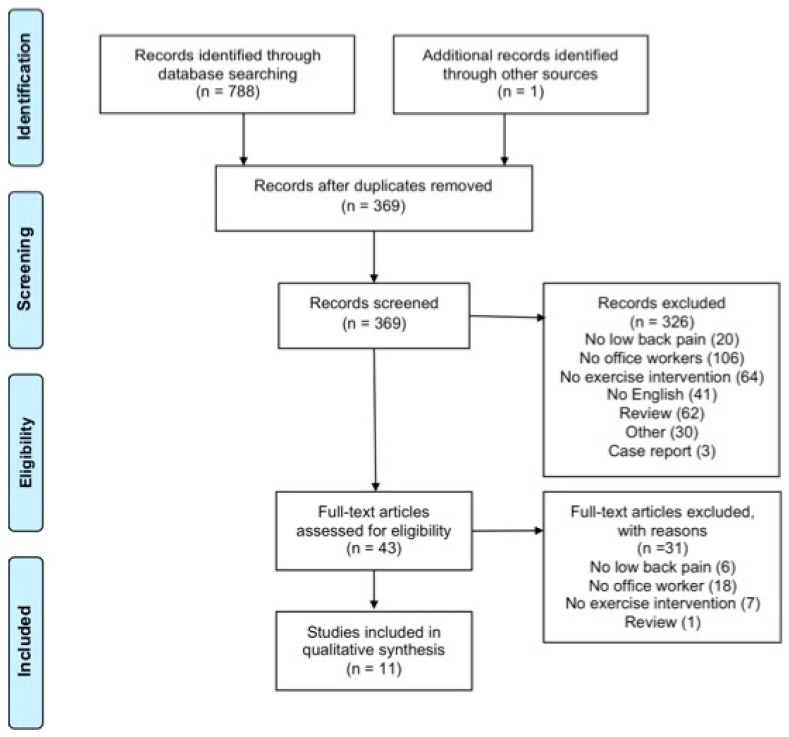
Flow chart.

Seven-hundred-and-eighty-eight studies were screened and 326 abstracts were read. Applying the inclusion and exclusion criteria, 11 articles were included in the systematic review. Figure 1 shows the exclusion process. Table 2 shows the quality assessments of the included studies. Seven studies were classified as high quality [15,16,17,20,22,23,24], and four as low quality [18,19,21,25]. Randomization was performed in eight studies [15,16,17,18,20,21,22,23], seven specified the similarity between groups at baseline evaluation [16,17,18,19,20,22,23], and nine the inclusion and exclusion criteria [15,16,17,20,21,22,23,24,25]. Six studies applied a blinding procedure [15,16,17,20,22,23], and two performed an “intention-to-treat” analysis [15,23]. Dropout ratio was reported in nine manuscripts [15,16,17,20,21,22,23,24,25], and in six the compliance [17,20,22,23,24,25]. Finally, three papers reported the timing of the outcome’s assessment [15,19,24] and three performed a follow-up evaluation [17,20,24].

The characteristics of the different protocols are summarized in Table 3. The sample sizes of the included studies ranged from 30 to 164 participants, aged 18 to 60 years old. Physical exercise programs lasted from 6 weeks to 12 months in terms of duration and were performed 1 to 5 days per week. Each single exercise lesson lasted from 10 to 60 min performed in a single session or split into 2–3 parts throughout the working day. Table 4 reports the results of the included studies.

### 3.1. Pain and Disability

All the included studies analyzed pain and disability before and after exercise [15,16,17,18,19,20,21,22,23,24,25]. The visual analogue scale (VAS) was used in six studies [15,17,20,22,24,25]. The Qigong exercise protocol performed for 6 weeks induced a significant reduction in pain after 1 week (−35%), with a higher value at the end of the program (−71%) [15]. Kim and colleagues [20] analyzed the VAS at rest and during movement. After 8 weeks of HE, they recorded a significant reduction in pain of 63.3% at rest and 56.7% during movement [20]. According to Kim et al. [20], home-based exercise induced a reduction of 42% after 8 weeks in the Pensri et al. study also [24]. Web-based exercise performed daily during work for 9 months produced an improvement in pain symptoms in 87% of office workers included in the study, while 13% reported no change [22]. In addition, Macedo et al. [25] found significant improvement after 8 months of exercise. No statistically significant changes were found in the other two studies [17,26]. The Roland and Morris Disability Questionnaire (RMDQ) was used in two studies [15,24]. Qigong induced a significant improvement after 5 weeks (−58.7%) [15], while no significant modifications were found after home-based exercise [24]. Shariat and colleagues [18] found a 40% reduction in LBP after 11 weeks of web-based exercise, evaluating participants with the Cornell Musculoskeletal Disorders Questionnaire. In another study, the authors found a reduction of 89.1% after 6 months of exercise [16]. Interestingly, after their web-based protocol, Habibi and colleagues [19] described a reduction in LBP incidence following 2.5 months of training, as well as pain duration after 1 month [21]. Eight weeks of home-based exercise improved the pain press threshold (PPT) of the quadratus lumborum (44%) and sacroiliac joint (42.6%) [20]. Positive outcomes were also found in the Del Pozo–Cruz et al. [22] investigation: following 9 months of web-based exercise, improvements in the Oswestry Disability Index were gathered in 37% of participants. Moreover, the authors displayed significant and clinically meaningful differences between the web-based exercise group and the control group (CG) in the total score of the Kneele Start Back Screening Tool (SBST) with particular modification in functional disability and fear avoidance [23].

### 3.2. Flexibility, ROM, and Muscular Strength

Four studies evaluated flexibility and ROM after different exercise protocols [15,17,18,20]. Six weeks of Qigong exercise induced a significant improvement in the lumbar flexion and extension (16.5%, 42.6%), lumbar rotation to left side (23.8%), and lumbar bending to right (14%) and left (19.2%) sides, whereas no significant difference was found in the lumbar rotation to the right side [15]. Ten weeks of exercise developed for neck and back pain induced significant improvement in trunk side bending [17]. After 8 weeks of home-based exercise intervention, Kim and colleagues [20] found significant improvement in trunk flexion and extension (45.1%, 44.6%). Moreover, the authors found a 51.9% and 43.5% improvement in the proprioception capacity to achieve the 20° of trunk flexion and 10° of trunk extension [20].

Two studies evaluated muscular strength [15,17]. Only Phattharasupharerk and colleagues [15] found significant improvement in core stability index (55.3%) after 6 weeks of Qigong. Suni and colleagues [17] found significant improvement in the static wall test after 10 weeks of exercise, only in female participants.

### 3.3. Quality of Life

Quality of life (QoL) was evaluated in three studies [15,17,22]. Del Pozo–Cruz and colleagues [22] used the European Quality of Life Questionnaire with 5 dimensions and 3 levels (EQ-5D-3L) finding significant improvements in 97.8% of participants who performed web-based exercise compared with the CG. In more detail, 69.6% improved in the mobility dimension, 34.8% improved in the self-care dimension, 52.2% in the pain and discomfort dimension, and finally, 37% showed a decrease in the anxiety and depression dimension [22]. In Suni et al. [17], the evaluation of QoL with a 36 item short-form health survey (SF-36) described significant improvements after 11 weeks of exercise. The authors [17] found significant improvements in physical role functioning, general health, and vitality, while significant differences among groups were found in physical functioning and bodily pain. Finally, the Srithanya stress scale (ST-5) recorded significant differences between qigong and ergonomic intervention after 6 weeks of intervention [15].

## 4. Discussion

This systematic review aimed to summarize the effectiveness of different types of exercise interventions to reduce LBP symptoms and improve muscle strength and flexibility of lower limbs and the trunk in office workers. Protocols were supervised and unsupervised, with exercise performed at the workplace (WE) and/or at home or with a web support, compared with sedentary groups (CGs), ergonomic intervention groups, educational program groups, or therapy intervention groups.

### 4.1. Effects of Physical Exercise on Pain and Disability

All the included studies analyzed the effects of exercise in different types of low backache, including sub-acute LBP [16,17,18,22,23], chronic non-specific LBP (CNLBP) [15,20,24], and pain without a specific classification [19,21,25]. A different intervention was applied in office workers with sub-acute LBP and only one study did not show significant reductions in LBP symptoms [17]. According to the authors, the lack of improvements was related to the exercise program focused on postural and movement control of neck and shoulders. Indeed, significant reductions in these two specific areas were found [17]. Moreover, it seemed that a different protocol duration reduced LBP in a different manner, from 63% after 8 weeks to 89% after 24 weeks [16]. Qigong practice combined with meditation reduces LBP in office workers with CNLBP. This effect could be ascribed by the improvements in muscle strength and flexibility; moreover, meditation seemed to reduce pain perception [27]. In fact, meditation activity can positively moderate the autonomic function inducing physiological modifications that involve cardio-circulatory function [27].

Only one investigation was focused on core muscle strengthening with the integration of TENS and hot pack therapies [20]. The results showed significant reductions of LBP at rest and during movement, while there was no significant modification for the control group which performed only TENS and hot pack treatments. For this reason, we speculate that the major effect in the reduction of LBP could be linked to the exercise effect, with respect to TENS and hot pack treatments [20].

Similar protocols were adopted in Shariat [16] and Macedo [25] studies which consisted of stretching and strengthening exercise training performed in the workplace. The only difference was the method to deliver the exercise protocol: in the first study, the employees had to perform the exercises following a video upload on their computer, while the second study was administered in the form of a supervised exercise training protocol. Exposures were similar for both investigations, with programs lasting 15 min carried out three times per week for 6 [16] and 8 [25] months, respectively. In both manuscripts, significant improvements in LBP were found, while the pain symptoms worsened in the CG. Despite the improvements, an interesting result was found in regard to the dropout ratio. In detail, Macedo and colleagues [25] reported that 48% of participants ended the exercise protocol, in contrast to Shariat and colleagues [16] who recorded a 95% rate of adherence to the exercise protocol. This discrepancy could be ascribed to personal reasons and/or different levels of motivation, such as organizational issues of the guest company linked to the production. As a simple example, workers may prefer not to be engaged in a work-out during their lunchbreak, or they could feel embarrassed to exercise in public or with colleagues [28]. The exercise protocol of Macedo and colleagues [25] consisted of sessions that were performed in pairs or in groups, while Shariat et al.’s [16] exercise protocols were administered following videos displayed on computer screens. It may be that the method to deliver exercise in the workplace was preferred in the latter study. It is also difficult to determine the reasons underlying these differences in dropout rates because the original modalities of participation were not completely explained in the aforementioned studies.

The other six studies used video support as an exercise training program. Low back pain symptoms after 11 weeks [18], 6 months [16], and 9 months [22,23] improved when comparing web-based groups with CG. Moreover, it seemed that bouts of 10–15 minutes of stretching exercises performed 3 to 5 day per week were sufficient for the management of LBP at workplace. Web-based exercises appeared innovative and potentially effective when applied alone. These potential benefits could be amplified in combination with ergonomics enhancements where possible [16]. Ergonomic modifications were the most frequent adaptation in the LBP prevention context with positive effects [16,19,21], but they were not easily applicable in all workstation. Indeed, costs have to be considered for ergonomic adaptations and reductions in environmental barriers, which may be critical for some facilities.

### 4.2. Effects of Physical Exercise on Flexibility, ROM, and Muscular Strength

Overall, the information and results indicated that the most effective exercise protocol should include a program to strengthen the abdominal region and lower limbs, in particular hamstrings. In fact, LBP was often paired by reduced lower-limb flexibility and limited muscle strength capacity in the lower body. Moreover, abdominal and leg muscular strength were negatively correlated with LBP intensity, and those parameters negatively influenced the activity of daily living [29].

Hoon Kim and colleagues developed a CORE program focused on the strengthening and stretching of trunk muscles, and high adherence (85.5%) induced significant improvements in flexibility and ROM of the trunk, such as a reduction in LBP [20]. Moreover, an interesting finding of this research was the significant improvement in proprioception capacity to achieve a specific trunk degree of flexion and extension [20]. Often, LBP was associated with proprioception impairment [30], and improvement may have reduced LBP.

The major improvement in trunk flexibility and abdominal strength seemed to be associated with qigong and meditation practice, also when compared with an educational program group [15]. In this study, participants performed 1 h of qigong exercise for 6 weeks at their workstation, and participants were encouraged to continue training also at home. The authors explained their results analyzing the characteristics of qigong, focusing on posture and breathing [15]. In fact, the slow dynamic movement associated with deep breathing helped relaxation and probably increased the muscle stretching capacity. Moreover, the ending postures of qigong are similar to specific exercise postures generally indicated to increase back flexibility [15].

The use of personal computers as an incentive to exercise in the workplace was applied by Shariat and colleagues [18]. They recorded a 15 min video clip with exercises that office workers had to perform three times per week. The program included stretching exercises for trunk, legs, and shoulders performed in sitting and standing positions. This protocol improved the ROM of hip and knees, in a small but significant manner. However, the CG worsened or did not changes their hip and knee ROM, and the difference among the two groups was statistically significant [18]. Due to the study’s design, the low sample size should have been increased, which may have led to a larger significant change in the results.

Another study was designed in two steps: The first 10 weeks of training were performed in private gyms under the supervision of a physiotherapist or a graduate in sports science. The second step was a set of 9 months where participants continued a personal exercise program at their homes. Interestingly, similar to the study design, a first initial improvement followed the supervised period of training for both flexibility and muscle strength, while a general maintenance was detected 9 months later. In other words, the supervised part of program was more effective than the self-managed part; however, the latter was sufficient to maintain the level of conditioning reached during the first part [17].

### 4.3. Effects of Physical Exercise on Quality of Life

Stress and anxiety of white-collar workers are frequently associated with high work demand that, in turn, is an associated risk factor for LBP [31]. For this reason, the introduction of specific exercise programs developed to restore specific body regions could reduce LBP symptomatology and stress, improving the QoL of the employees. The included studies of this review analyzed QoL introducing exercise programs in the workplace or at home, showing significant improvements in QoL. The qigong and meditation protocol was able to induce significant reductions in stress-related outcomes in office workers with chronic non-specific low back pain (CNLBP) [15]. Also, in sub-acute LBP white-collar workers, QoL improved after exercise. The web-based exercise program was performed at the workstation, with a video designed to remind workers of the correct posture, followed by specific exercises to strengthen and stretch postural muscles [22]. Even if daily practice for 9 months did not significantly improve the QoL of office workers, these domains showed significant differences compared to the CG. Moreover, the authors found a strong and clinical meaningful relationship between pain/discomfort and mobility QoL dimensions with the changes in back pain. This suggested the efficacy of this type of intervention from a preventive perspective. Finally, the high adherence to exercise (88%) suggested the feasibility of this type of intervention in a workplace setting [22]. According to the flexibility and muscle strength evaluation, QoL also improved after 10 weeks of supervised exercise and was maintained in the follow-up evaluation after 9 months [17]. This finding agreed with other research that found the largest improvement in the supervised exercise program; however, more investigation is necessary to enhance its efficacy, especially in the addition other physiological and psychological parameters to allow deeper analyses [32].

### 4.4. Limitations

This review presents some limitations. First off, exercise protocols were largely different, and in some studies, they were integrated with other activities such as meditation and physiotherapy treatments. Therefore, it is difficult to ascribe and precisely quantify the magnitude of changes in LBP symptomatology due to the exercise. Secondly, adherence ratios were not reported in all the included investigations and only two papers reported the intention-to-treat analysis. Both parameters are extremely important to understand the effectiveness of the intervention, especially in an environment that is not well known as a place to workout. Finally, the participants had different types of LBP and it is difficult identify the better type of protocol for sub-acute LBP or CNLBP.

## 5. Conclusions

The results of this systematic review showed that exercise programs in the workplace were effective and able to reduce LBP symptoms in office workers, improving muscle strength, flexibility, and increasing their quality of life. The manuscripts’ data reported no side effects; however, this is not sufficient to allow for the statement of the complete safety of these protocols. The best improvements were achieved in supervised protocols and web-based protocols. Probably, exercising following videos or under supervision support and guarantee the correct execution of the different exercises and facilitate their efficacy. We strongly encourage the development and broadcast of simple videos together with the promotion of exercise sessions handled by an exercise specialist, since these exercise modalities appeared to be able to generate meaningful improvements. Effects can also be produced with small duration sessions during the working day, with only 10–15 minutes of adapted exercise to be performed 3–5 days per week. Additionally, specific exercise prescriptions from an occupational physician may be highly useful as preventive actions or as concrete tools to reduce symptomatology for LBP office workers. Future research will be needed to confirm the results of this review to improve the lack of knowledge in terms of adherence and intensity, such as follow-up evaluations to determine the maintenance of training modifications.

## Figures and Tables

**Table 1:** Participants, interventions, comparators, outcomes, study (PICOS) model.

PICOS	Details
Participants	Office workers with LBP symptoms
Interventions	Supervised or non-supervised exercise protocol, performed at home or in the workplace
Comparative factors	Exercise intervention for LBP management
Outcomes	Primary outcomes: LBP symptoms Secondary outcomes: flexibility and ROM of the trunk, muscular strength of the trunk, QoL
Study designs	Pilot study, RCT, no-RCT, exploratory study, Randomized pilot trial

LBP: low back pain; ROM: range of motion; QoL: quality of life; RCT: randomized, controlled trial.

**Table 2:** Quality assessments of intervention studies.

Citation	Randomization Procedure	Similarity of Study Groups	Inclusion or Exclusion Criteria	Dropouts	Blinding	Compliance	Intention-to-Treat Analysis	Timing of Outcomes Assessment	Follow-up	Results
Phattharasupharerk S. et al. (2018) [15]	+	−	+	+	+	−	+	+	−	6/9
Shariat A. et al. (2018) [16]	+	+	+	+	+	-	−	−	−	5/9
Suni J.H. et al. (2017) [17]	+	+	+	+	+	+	−	−	+	7/9
Shariat A. et al. (2017) [18]	+	+	−	−	−	−	−	−	−	2/9
Habibi E. et al. (2015) [19]	−	+	−	−	−	−	−	+	−	2/9
Kim T.H. et al. (2015) [20]	+	+	+	+	+	+	−	−	+	7/9
Mehrparvar A. H. et al. (2014) [21]	+	−	+	+	−	−	−	−	−	3/9
del Pozo-Cruz B. et al. (2013) [22]	+	+	+	+	+	+	−	−	−	6/9
del Pozo-Cruz B. et al. (2012) [23]	+	+	+	+	+	+	+	−	−	7/9
Pensri P. et al. (2012) [24]	−	−	+	+	−	+	−	+	+	5/9
Macedo A.C. et al. (2011) [25]	−	−	−	+	−	+	−	−	−	2/9

**Table 3:** Characteristics of the studies.

Study	Subjects	Grouping	Training Modality, Program and Intensity	Duration and Frequency
Phattharasupharerk S. et al. (2018) [15]	N: 72 Age: 20–40 years old Chronic LBP	EG (36) EdG (36)	EG: Qigong protocol Based on static and dynamic posture, meditation/imagination, and breathing exercise. Week 1: 25 min of static qigong, 5 min of dynamic qigong, 2 min of acupressure at acupoint GV20, 10 min of Wu Chi meditation. Week 2: 28 min of static qigong, 4 min of acupressure at acupoint K11, 15 min of Wu Chi meditation. Week 3: 28 min of static qigong, 5 min of dynamic qigong, 4 min of acupressure at acupoint LI4, 15 min of Wu Chi meditation Week 4: 28 min of static qigong, 5 min of dynamic qigong, 4 min of acupressure at acupoint PC6, 15 min of Wu Chi meditation. Week 5: 28 min of static qigong, 5 min of dynamic qigong, 4 min of acupressure at acupoint ST36, 15 min of Wu Chi meditation. Week 6: 4 min of static qigong, 10 min of dynamic qigong, 4 min of acupressure at acupoint HT7, 15 min of Wu Chi meditation. EdG: Education protocol General advice on managing LBP in order to reduce it, and the recommendation to stay active.	6 weeks 60 min 1 d/w At home every day Adh: n.r.
Shariat A. et al. (2018) [16]	N: 142 (47 M; 95 F) Age: 20–50 years old Subacute LBP	WBG (43) EgG (37) WB+EgG (34) CG (28)	WBG: stretching protocol Office-based stretching exercises adopted from McKenzie’s exercises, William’s exercises, and ACSM guidelines. The exercises were performed constant, controlled, and slow. Tension was progressively increased to the end of the joint’s ROM until the mild discomfort point is touched. 10 repetitions (or last for a period of 10–15 sec) and 3 sets (with a rest of 60–90 sec. Ergonomic protocol Total workplace occupational safety and health and ergonomic intervention (chair height and working desk, sitting posture, distance and level between the eyes and the monitor).	6 months 3 d/w 10–15 min Adh: n.r.
Suni J.H. et al. (2017) [17]	N: 143 Age 30–50 years old Subacute LBP	EG (75) CG (68)	Warm-up: 10 min of aerobic exercise. Main part: 10 functional flexibility exercises, 4 strength, and 5 core exercises. Cool-down: 10 min of stretching.	EG: 10 weeks 2 d/w 60 min Adh: 67%
Shariat A. et al. (2017) [18]	N: 40 Age: mean 28 years old	WBG (20) CG (20)	13 office-based stretching exercises. Each exercise was designed to have slow, controlled, and constant movements. Tension was progressively increased to the end of the joint’s ROM until the mild discomfort point is touched. The entire set of exercise was performed for 3 times with a rest of 60–90 sec between sets. Week 1–2: learn and practice. Week 3–5: 10 sec for each exercise. Week 6–8: 20 sec for each exercise. Week 9–11: 30 sec for each exercise.	11 weeks 10–15 min 3 d/w Adh: n.r.
Habibi E. et al. (2015) [19]	N: 75 (52 M; 23 F) Age: mean 41.2 years old	EgG (25) EG (25) WBG (25)	Ergonomic protocol Different courses based on educational needs. The objectives were understanding office ergonomics principles, self-evaluation of workplace conditions, and arranging and organizing personal workspace. EG: water protocol Water exercise to strengthen the muscles around the spine. SG: web-based exercise Exercises during work time. They were designed for short periods of time, and for many of them, there was no need to get up and stand. At regular intervals the app reminded the user to exercise.	EgG and WBG 2.5 months EG 2.5 months 2–3 d/w 20 min Adh: n.r.
Kim T.H. et al. (2014) [20]	N: 53, F Age: 20–40 years old Chronic LBP	HG (27) TG (26)	HG: Exercise protocol + TENS Warm-up Main part: isometric contraction of core muscles, including internal/external oblique, rectus abdominis, and erector spinae muscles. Cool-down: slow and controlled movement, controlling their breathing. TG: TENS treatment 20 min of TENS and 15 min of hot-pack treatment.	8 weeks 30 min exercise (only HG) 20 min TENS 15 min hot pack 5 d/w Adh: 85.5%
Mehrparvar A. H. et al. (2014) [21]	N: 164 (81 M; 83 F) Age: mean 38 years old	EgG (83) WBG (81)	Ergonomic protocol Change in desk placement, seat height, position of keyboard, mouse and monitor, following OSHA VDT workstation checklist. SG: Exercise protocol One training session to learn exercise. Stretching of neck, shoulder, writ, back, and low back.	1 months 15 min 2 times/day, every day Adh: n.r.
del Pozo-Cruz B. et al. (2013) [22] del Pozo-Cruz B. et al. (2012) [23]	N: 90 (12 M; 78 F) Age: mean 46 years old Subacute LBP	WBG (46)CG (44)	Postural stability muscles (abdominal, lumbar, hip and thigh muscles) exercise to strength, flexibility, mobility, and stretching. Mobility exercises: large movements of the joints associated with the postural stability muscles. Flexibility exercises: static work methodology. Strengthening exercises: shortening and stretching motion that progressively changed in speed (1:1, 1:2, 1:3, 2:1, 3:1) combined with slight isometric contractions of the muscles involved in the exercises. Stretching exercises: moderate stretching of the muscles involved in the session.	9 months 11 min 5 d/w Adh: 92%
Pensri P. et al. (2012) [24]	N: 30 (6 M; 24 F) Age: 18–60 years old Chronic LBP	HG (30)	Brief education regarding LBP and home-based exercise protocol. Home-based exercises were categorized in core stability, stretching, and mobility. Each exercise was performed for 5–15 sec and repeat 5 times per set. Sometimes they received treatment including hot packs, lumbar packs, lumbar traction, and/or electrotherapy.	8 weeks 3 t/d Adh: 33 day of 40
Macedo A.C. et al. (2011) [25]	N: 40 Age: 40.8 years old	EG (29) CG (21)	Stretching exercises for the body parts most affected by pain complaints. Playful and recreational activities. Massage with physiotherapy ball and exercises with Pilates balls. Relaxation exercises/stretches on an individual basis, in pairs and in groups performed with background music.	8 months 15 min 3 d/w Adh: 48%

*N*: number of subjects; M: male; F: female; d/w: day/week; Adh: adherence; n.r.: not reported; EG: exercise group; EdG: education group; WBG: web-based exercise group; CG: control group; EgG: ergonomic group; HG: home exercise group; TG: therapy group; ACSM: American College of Sport Medicine; TENS: transcutaneous electrical nerve stimulation; LBP: low back pain; OSHA VDT: Occupational Safety and Health Administration for visual display terminal

**Table 4:** Study results.

Study	Group Comparison	Results
Phattharasupharerk S. et al. (2018) [15]	EG versus EdG	*Pain and disability* VAS (sc): ↑EG *; ↓EdG; ** RMDQ (sc): ↑EG *; ↓EdG; ** Quality of life ST-5 (sc): ↑EG; ↓EdG; ** Flexibility, ROM and muscular strength Lumbar flexion (°): ↑EG *; ↓EdG; ** Lumbar extension (°): #; ↑EG *; =EdG; ** Lumbar rotation R (°): ↑EG; ↓EdG; ** Lumbar rotation L (°): #; ↑EG *; =EdG; ** Lumbar bending R (°): ↑EG *; =EdG; ** Lumbar bending R (°): ↑EG *; =EdG; ** Core stability index (mmHg * sec): ↑EG *; ↓EdG; **
Shariat A. et al. (2018) [16]	WBG versus EgG	*Pain and disability* LB CMDQ (sc): ↑EG *; ↑EgG *
	WBG versus WB+EgG	*Pain and disability* LB CMDQ (sc): ↑EG *; ↑E+EgG *
	WBG versus CG	*Pain and disability* LB CMDQ (sc): ↑EG *; =CG; **
	EgG versus WB+EgG	*Pain and disability* LB CMDQ (sc): ↑EgG *; ↑E+EgG *
	EgG versus CG	*Pain and disability* LB CMDQ (sc): ↑EgG *; =CG
	WB+EgG versus CG	*Pain and disability* LB CMDQ (sc): ↑E+EgG *; =CG; **
Suni J.H. et al. (2017) [17]	EG versus CG	*Pain and disability* LB VAS (sc): ↑EG; ↑CG LBP frequency (sc): ↑EG; ↑CG LB strain after work (sc): ↑EG; ↑CG *Quality of life* SF-36-PF: ↑EG; ↑CG; ** SF-36-RP: ↑EG *; ↑CG SF-36-BP: ↑EG; ↑CG; ** SF-36-GH: ↑EG *; ↑CG SF-36-VT: ↑EG *; ↑CG SF-36-SF: n.r. SF-36-RE: ↑EG; ↓CG SF-36-MH: n.r. *Flexibility, ROM and muscular strength* Trunk-side bending (cm): ↑EG *; ↑CG; ** Static wall squat test (sec): ↑EG *; ↑CG; **
Shariat A. et al. (2017) [18]	WBG versus CG	*Pain and disability* LB CMDQ (sc): ↑WBG *; =CG; ** Flexibility, ROM and muscular strength Hip R (°): ↑WBG; ↓CG; ** Hip L (°): ↑WBG; =CG; ** Knee R (°): ↑WBG; =CG; ** Knee L (°): ↑WBG; =CG; **
Habibi E. et al. (2015) [19]	EG versus EgG	*Pain and disability* LBP incidence (%): ↑EG *; ↑EgG *
	EG versus WBG	*Pain and disability* LBP incidence (%): ↑EG *; =WBG
	EgG versus WBG	*Pain and disability* LBP incidence (%): ↑EgG *; =WBG
Kim T.H. et al. (2015) [20]	HG versus TG	*Pain and disability* VAS at rest (mm): ↑HG *; ↑TG; ** VAS during movement (mm): ↑HG *; ↑TG; ** PPT quadratus lumborum (Kg/cm^2^): ↑HG *; ↑TG; ** PPT sacroiliac joint (Kg/cm^2^): ↑HG *; ↑TG; ** Flexibility, ROM and muscular strength Active ROM trunk flexion (°):↑HG *; ↑TG; **Active ROM trunk extension (°):↑HG *; ↑TG; ** Propioception at 20° flexion (°):↑HG *; ↑TG; ** Propioception at 10° extension (°):↑HG *; ↑TG; **
Mehrparvar A.H. et al. (2014) [21]	WGB versus EgG	*Pain and disability* Pain frequency: ↑WBG *; ↑EgG *; **
Del Pozo–Cruz B. et al. (2013) [22]	WBG versus CG	*Pain and disability* VAS (%): ** ODI (%): ** SBST (%): ** Quality of life EQ-5D-3L (%): ** Mobility: ** Self-care: ** Daily task: Pain/discomfort: ** Anxiety/depression: **
Del Pozo-Cruz B. et al. (2012) [23]	WBG versus CG	*Pain and disability* SBST, total (sc): ↑WBG *; =CG; ** Item1 - Bothersomeness: ↑WBG; ↓CG Item2 - Referred leg pain: ↑WBG; ↓CG Item3 - Co-morbid pain: ↑WBG; ↑CG Item4 - Fear avoidance: ↑WBG; ↑CG; ** Item5 - Functional disability: ↑WBG; ↓CG; ** Item6 - Functional disability: ↑WBG; ↑CG; ** Item7 - Catastrophizing: ↑WBG; ↑CG Item8 - Anxiety: ↑WBG; ↓CG Item9 - Depression: ↑WBG; ↑CG Low risk of chronicity (%): ↑WBG; ↓CG ** Medium risk of chronicity (%): ↑WBG; ↓CG High risk of chronicity (%): ↑WBG; ↓CG
Pensri P. et al. (2012) [24]	HG	*Pain and disability* VAS (sc): ↑ * Backache Index (sc): ↑
Macedo AC et al. (2011) [25]	EG versus CG	*Pain and disability* VAS – L lumbar zone (mm): ↑EG *; ↓CG; ** VAS – R lumbar zone (mm): ↑EG *; ↓CG; **

*p* < 0.05; * intra-group difference; ** between group difference; # significant difference at baseline; ↑ improvement; ↓ worsening; = no change; EG: exercise group; EdG: education group; EgG: ergonomic group; CG: control group; WBG: web-based exercise group; HG: home exercise group; TG: therapy group VAS: visual analogue scale; RMDQ; Roland and Morris Disability Questionnaire; ST-5: Srithanya Stress Scale; R: right; L: left; LB: low back; CMDQ: Cornell Musculoskeletal Disorders Questionnaire; SF-36: 36 item short-form health survey; PF: physical functioning; RP: physical role functioning; BP: bodily pain; GH: health perception; VT: vitality; SF: social functioning; RE: emotional role functioning; MH: mental health; LBP: low back pain; PPT: pain pressure threshold; ODI: Oswestry disability index; SBST: Keele Start Back Screening Tool; EQ-5D-3L: European Quality of Life Questionnaire, 5 dimensions, 3 levels.
